# Medical students’ career preferences in Bangladesh

**DOI:** 10.1186/s12909-024-05050-9

**Published:** 2024-01-23

**Authors:** Mohammad Azmain Iktidar, Md Muid Sakib, Ummi Rukaiya Munni, Fahmida Hoque Rimti, Renessa Yousuf, Koushik Majumder, Tirtha Saha, Farhat Lamisa Golpo, Md Samee U Sayed, Sabrina Monsur, Asadul Al Galib, Md Kamran Hossain, Sigma Alam Shupti, Noshin Nawar, Sudeshna Mazumder, M. Tasdik Hasan

**Affiliations:** 1 Directorate General of Health Services, Dhaka, Bangladesh; 2 Department of Public Health, North South University, Plot # 15, Block # B, Bashundhara R/A, 1229 Dhaka, Bangladesh; 3 School of Research, Chattogram, Bangladesh; 4 Chattogram Medical College, 57 K.B. Fazlul Kader Road, 4203 Chattogram, Bangladesh; 5 International Centre for Diarrhoeal Disease Research, Dhaka, Bangladesh; 6 Rajshahi Medical College and Hospital, 6000 Rajshahi, Bangladesh; 7 Dhaka Medical College Hospital, 1000 Ramna, Dhaka Bangladesh; 8 Sir Salimullah Medical College and Mitford Hospital, 1100 Mitford, Dhaka Bangladesh; 9 IBN Sina Medical College and Hospital, 1216 Kallyanpur, Dhaka Bangladesh; 10 Mymensingh Medical College and Hospital, Chorpara, Mymensingh, Bangladesh; 11 University of Chittagong, Chattogram, Bangladesh; 12 Action Lab, Department of Human Centred Computing, Faculty of Information Technology, Monash University, Melbourne, Australia; 13 Department of Public Health, State University of Bangladesh (SUB), Dhaka, Bangladesh; 14 Public Health Foundation, Bangladesh (PHF, BD), Dhaka, Bangladesh

**Keywords:** Career Preference, Bangladesh, Medical Student

## Abstract

**Aim:**

This study aimed to investigate the career preferences among Bangladeshi medical students, identify the factors that influence their present choices, and additionally report the role of gender and academic year behind their decisions.

**Methods:**

This cross-sectional study conducted in Bangladesh from August 2022 to April 2023 included 801 medical students conveniently selected from medical colleges in eight divisions. Data were collected using a web-based survey and analysed using STATA version 16.0. Statistical tests included the Kolmogorov-Smirnov test, arithmetic mean, standard deviation, frequency, and Kruskal-Wallis H test. The response rate was 94.6%, and the CHERRIES guideline was followed for reporting the results.

**Result:**

The majority of the participants were female (64.42%) and under the age of 23 years (58.8%). The study revealed that medicine was the preferred career choice for the majority of students (65%), with surgery being the most popular first choice (30.21%) among them. Female medical students showed a significantly higher preference for gynaecology & obstetrics (*p* < 0.001), while male students had a significantly higher tendency to choose general practice (*p* = 0.002). There is a significant gender difference (*p* < 0.05) in the career preference factors, including professional prestige, role model influence, easy money, family time, promotion opportunities, income for lifestyle, and research opportunities. Academic year differences were also observed, with increasing interest in medicine and public health (*p* < 0.001), a decrease in interest in surgery (*p* < 0.001), and a decline in preference for non-medical careers as students progressed through their MBBS life (*p* < 0.05).

**Conclusion:**

Overall, medicine was the most popular speciality choice, however, male students preferred general practice and female students preferred gynaecology and obstetrics more. Personal passion, opportunities for contribution to society, professional prestige, having a direct dealing with patients, and income will allow an enjoyable lifestyle were the most important factors in the choice of their career.

**Supplementary Information:**

The online version contains supplementary material available at 10.1186/s12909-024-05050-9.

## Introduction

According to *Bangladesh Economic Review 2022*, Bangladesh critically suffers from both a shortage and unequal geographic distribution of physicians with only one physician per 1,724 population [1]. It has also been observed that health workers are concentrated in urban secondary and tertiary hospitals by choice, although 70% of the population lives in the rural areas [2]. Career choices of medical students significantly influence the workforce balance between different medical specialities [3] and without an appropriate workforce balance equitable healthcare service cannot be ensured [4].

Medical education at the undergraduate level in Bangladesh is overwhelmingly stressful [5, 6] and lasts for five years with an additional year of clinical internship [7]. At the end of their time in medical school, students are required to make a significant choice about the area of medical science in which they intend to practice. It denotes a considerable shift in the education that these students get, moving away from an all-encompassing exposure to a number of medical specializations and toward a more focused study in a particular field of medical practice [8]. Student choice serves as the jumping-off point for the career selection process, which is the consequence of a complex interplay between student expectations, department expectations, and competition for available places [9]. Previous studies have revealed that the choice of career made by medical students is critical to the upkeep of sufficient medical professionals and the preservation of a balanced expansion within the medical system [8, 10].

To understand the factors influencing the career paths chosen by medical student, several studies have examined personality traits and other key elements, such as income, lifestyle, and intellectual substance. Yang et al. found that academic interests, skills, and controllable lifestyles were the top three factors influencing medical students’ career choices, based on a comprehensive analysis of 75 studies from various populations [11]. Gender also plays a significant role in specialty selection, with males typically leaning towards surgery and females towards obstetrics and gynaecology [12]. Lifestyle factors are also critical in decision-making, as a study revealed that 36% of women and 45% of men prefer specializations with a controllable lifestyle [13].

However, there is a dearth of research reflecting the kinds of medical specialties that Bangladeshi medical students are interested in pursuing. In 2011, Ahmed et al. conducted the only study in this area using a sample size of 132 participants drawn from only two medical colleges in Dhaka, Bangladesh [14]. After ten years, when approximately 10,000 students are getting admitted into 115 medical institutions each year [15], it begs the question of whether or not the prior conclusion can be generalized to cover a larger population. Therefore, the main objective of this research is to investigate the contemporary career preferences among Bangladeshi medical students, identify different factors that influence their preference, and evaluate the role of gender and academic year behind their decisions. By accomplishing these objectives, this study aims to provide valuable insights into the career choices and motivations of medical students in Bangladesh, which can be used to inform future policies and interventions aimed at improving the country’s healthcare system.

## Methods

### Study design & study participants

This cross-sectional study was conducted in Bangladesh between August 2022 and April 2023. The research enrolled 801 medical students who were conveniently selected from medical colleges located in eight divisions of Bangladesh. To be eligible for the study, participants were required to satisfy the following criteria: (1) reside in Bangladesh, (2) be current medical students, and (3) provide informed consent.

### Pilot study

A pilot study was undertaken before the full survey. The survey questionnaire was disseminated to a sample of 40 students enrolled in a medical college, whereby every third eligible student at the institution was selected to receive the questionnaire. Based on the comments from the pilot study, the survey questionnaire was created. Some questions were updated based on face validity to improve the clarity and accuracy of the phrasing. The survey’s content validity was established through the evaluation of two medical college teachers who conducted an independent review. The reliability and internal consistency of the questionnaire were determined using Cronbach’s alpha coefficient (0.88).

### Instrument and measurement

The data collection process involved the utilization of a semi-structured and self-reported questionnaire, which was accompanied by an electronic informed consent form. The questionnaire comprised three sections, namely background characteristics, career choice, and factors influencing the participants’ career choice. The *background characteristics* section had 11 questions which included age, gender, religion, marital status division of residence, type of medical college (government/private), medical college location, academic year, parent’s highest educational status, any first-degree doctor relative, monthly family income (in BDT), and whether any supplementary examinations (when a student fails a subject, he/she has to take supplementary examination) taken. The *career choice* section had 3 questions, first, second and third career choice. Each questions had the following options: general practice (provide comprehensive primary healthcare to individuals and families), medical administration (management and coordination of healthcare facilities and services), medicine specialty (focus on the diagnosis and non-surgical treatment of various medical conditions), surgical specialty (specialize in performing surgical procedures to treat a wide range of medical conditions), gynaecology & obstetrics specialty (focus on women’s reproductive health, addressing issues such as reproductive system disorders and pregnancy-related concerns, childbirth, and postpartum care), basic medical science (encompasses areas like anatomy, physiology, biochemistry, pathology, microbiology and pharmacology), preventive & social medicine/public health (concentrate on preventing diseases and promoting health at the community and population levels), research (involve in the systematic investigation of medical phenomena to advance scientific knowledge), undecided, non-medical career, and other. Non-medical career choice was an open question, where many responded Bangladesh Civil Service (BCS) as their career choice. BCS has 26 cadre options, divided into two categories: general and technical. Any Bangladeshi graduate can apply for general cadres (i.e., BCS-Foreign Affairs, BCS-Administration, BCS-Police etc.). Individuals with professional degrees can apply in technical cadres. For example, BCS (Health) is a technical cadre for doctors. Finally, *factors influencing the participants’ career choice* section had 22 questions with a five-point Likert-type scale (1 = strongly disagree, 2 = disagree, 3 = neutral, 4 = agree, and 5 = strongly agree).

The full questionnaire (supplementary file 1) was then entered into *Google Forms* for online distribution without any item randomization and validated for usability and technical functionality. The form contained 37 questions spread across three pages. The mandatory items were denoted by a red asterisk and a corresponding non-response option was provided. Participants were afforded the opportunity to review and modify their responses, if deemed necessary, by utilizing the back button. To avoid duplicate entries, the survey was not presented again after the user had completed it.

### Data collection

Potential participants were reached through convenience sampling by nine research assistants who had received training and thorough information about the project. After confirming that the participants satisfied the eligibility requirements and gave their consent, a link to a closed web-based survey made with Google Forms was delivered through Facebook message, email, or SMS. There was no other form of promotion or advertising for the survey. Of the 847 eligible participants who agreed to participate, 801 participants completed the entire questionnaire (completion rate: 94.6%), and incomplete questionnaires were excluded from the analysis.

### Statistical analysis

We used Stata (version 16; StataCorp, College Station, TX, USA) for data analysis. A histogram, a normal Q-Q plot, and the Kolmogorov-Smirnov test were used to check for normality in continuous data. For quantitative data, the arithmetic mean and standard deviation were utilized as measures of central tendency and dispersion, respectively. Utilizing frequency and relative frequency, categorical data were condensed. Pearson’s Chi-square test was used to examine the relationship between career choice and independent variables such as socio-demographics. The mean attitude scores from a five-point Likert scale were compared using the non-parametric Mann-Whitney test and Kruskal-Wallis H test. The Checklist for Reporting Results of Internet E-Surveys (CHERRIES) requirements were followed throughout the reporting process [16].

## Results

Among 801 medical students in different medical colleges in Bangladesh, future career choices/ preferences and the factors associated with these choices have been estimated.

### Socio-demographic

Table 1 demonstrates the sociodemographic information of the study participants. The mean age of the participants was 21.95 (± 1.80) years. About 516 (64.42%) students were female. The majority of the students were from Dhaka (340; 42.45%). The highest proportion (592; 73.91%) of the students were from government/ public medical colleges and most (384; 48%) of the colleges were located in Dhaka. Two-hundred and fifty (31.21%) study participants were 3rd-year medical students. Most of the study participants were Muslim (691; 87.47%) and unmarried (752; 95.43%). About 42.2% of the student’s parents obtained a master’s degree. Maximum (542; 67.67%) study participants reported they had no first-degree doctor relatives. The largest proportion of the students (646; 80%) of students had no history of appearing in supplementary examinations. About 212 (26.47%) participants reported a monthly income of more than 60,000 BDT (Table 1).

**Table 1:** Background information of the study participants (*n* = 801)

Variable	Frequency	Percentage
**Age (in years), mean ± SD**	21.95 ± 1.80	
**Age Category**		
< 23 years	471	58.8
≥ 23 years	330	41.2
**Gender**		
Female	516	64.42
Male	285	35.58
**Division wise Residence**		
Dhaka	340	42.45
Chattogram	165	20.6
Rajshahi	103	12.86
Khulna	79	9.86
Mymensingh	33	4.12
Barisal	30	3.75
Sylhet	51	6.37
**Medical College Type**		
Government	592	73.91
Private	209	26.09
**Medical College Location**		
Dhaka	384	48
Chattogram	152	19
Rajshahi	86	10.75
Mymensingh	102	12.75
Other	76	9.5
**Academic Year**		
1st	146	18.23
2nd	81	10.11
3rd	250	31.21
4th	192	23.97
5th	132	16.48
**Religion**		
Islam	691	87.47
Sanatan/Hindu	86	10.89
Buddhism	7	0.89
Christianity	6	0.76
**Marital Status**		
Unmarried	752	95.43
Ever Married	36	4.57
**Parent’s Highest Educational Status**		
Illiterate	5	0.62
Primary	36	4.49
SSC	74	9.24
HSC	124	15.48
Bachelor	200	24.97
Masters	338	42.2
Doctoral	24	3
**First-degree doctor relative**		
No	542	67.67
Yes	259	32.33
**Monthly Family Income (in BDT)**		
< 15,000	75	9.36
15,000–29,999	175	21.85
30,000–44,999	208	25.97
45,000–59,999	131	16.35
> 60,000	212	26.47
**Supplementary Examination**		
No supplementary examination	646	80.65
1	78	9.74
2 or more	77	9.61

BDT, Bangladeshi Taka ((1 USD = 106 BDT); SD, standard deviation

### Distribution of future career choices

**Fig. 1:**
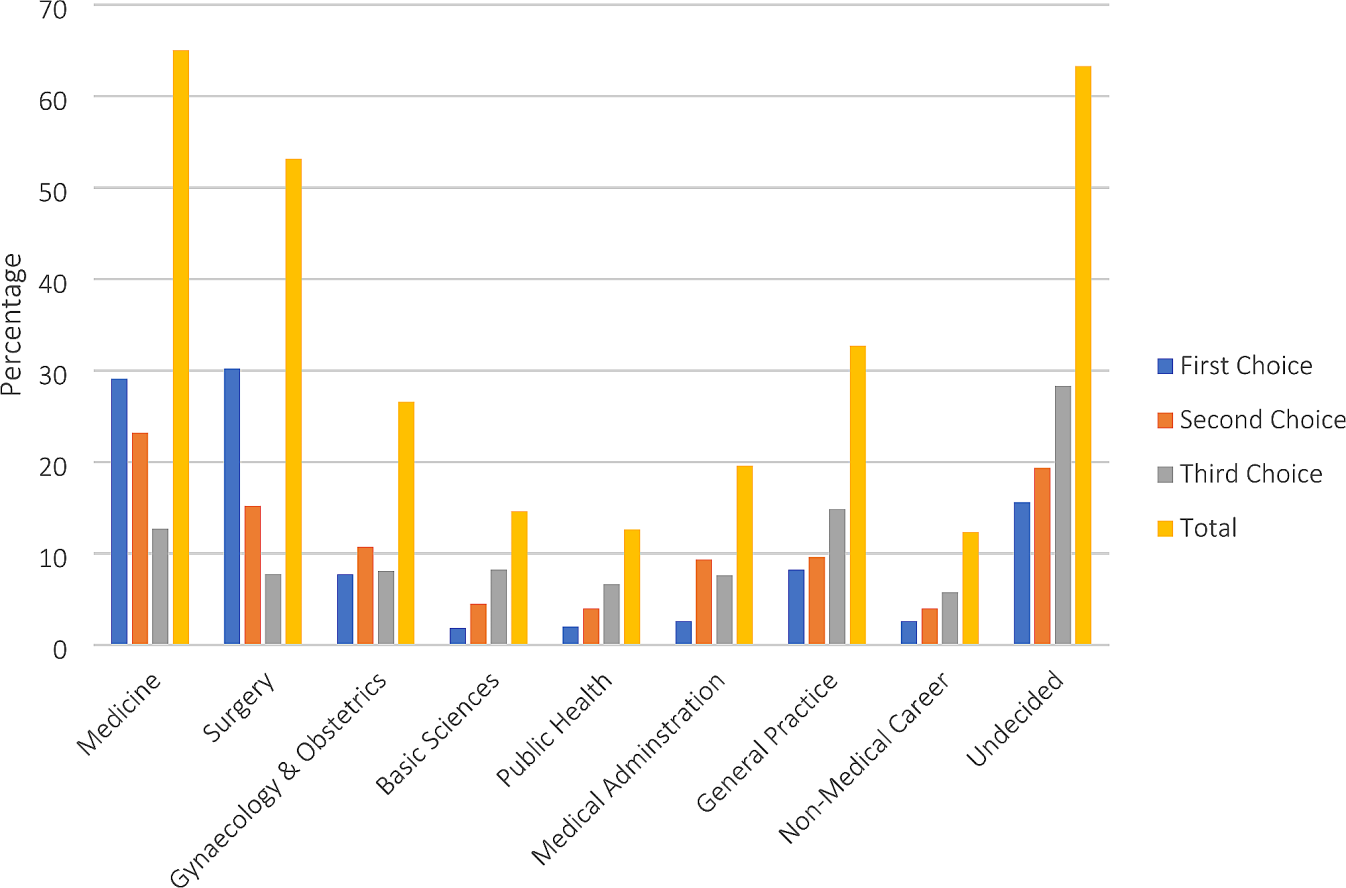
The distribution of career preferences divided into a first, second, and third choices among the medical students of Bangladesh during the 2022 academic year

The career preferences of the participant medical students are depicted in Fig. 1 according to their first, second, and third choices. Overall, the majority of the students inclined to Medicine as their future specialty and was chosen by about 65% of the participants. However, Surgery was the most popular first choice and was considered by 30.21% of students. On the other hand, the least preferred career choices were basic sciences, public health, medical administration, and non-medical career. However, a significant number (63.3%) of participants had not fixed their career choices at this point of their academic period.

### Non-medical career choices

Out of 801 students, 296 responded that they would follow a non-medical career path if they get the opportunity (Table 2). The most (105; 13.11%) preferred option was BCS Administration.

**Table 2:** Non-medical career choice of medical students (*n* = 296)

Non-medical Career Choice	Frequency	Percentage
BCS (Administration Cadre)	105	35%
BCS (Any General Cadre)	75	25%
Entrepreneur	41	14%
BCS (Foreign Cadre)	19	6%
BCS (Police Cadre)	10	3%
Other	46	16%

BCS, Bangladesh civil service

### Gender differences in career choices

**Fig. 2:**
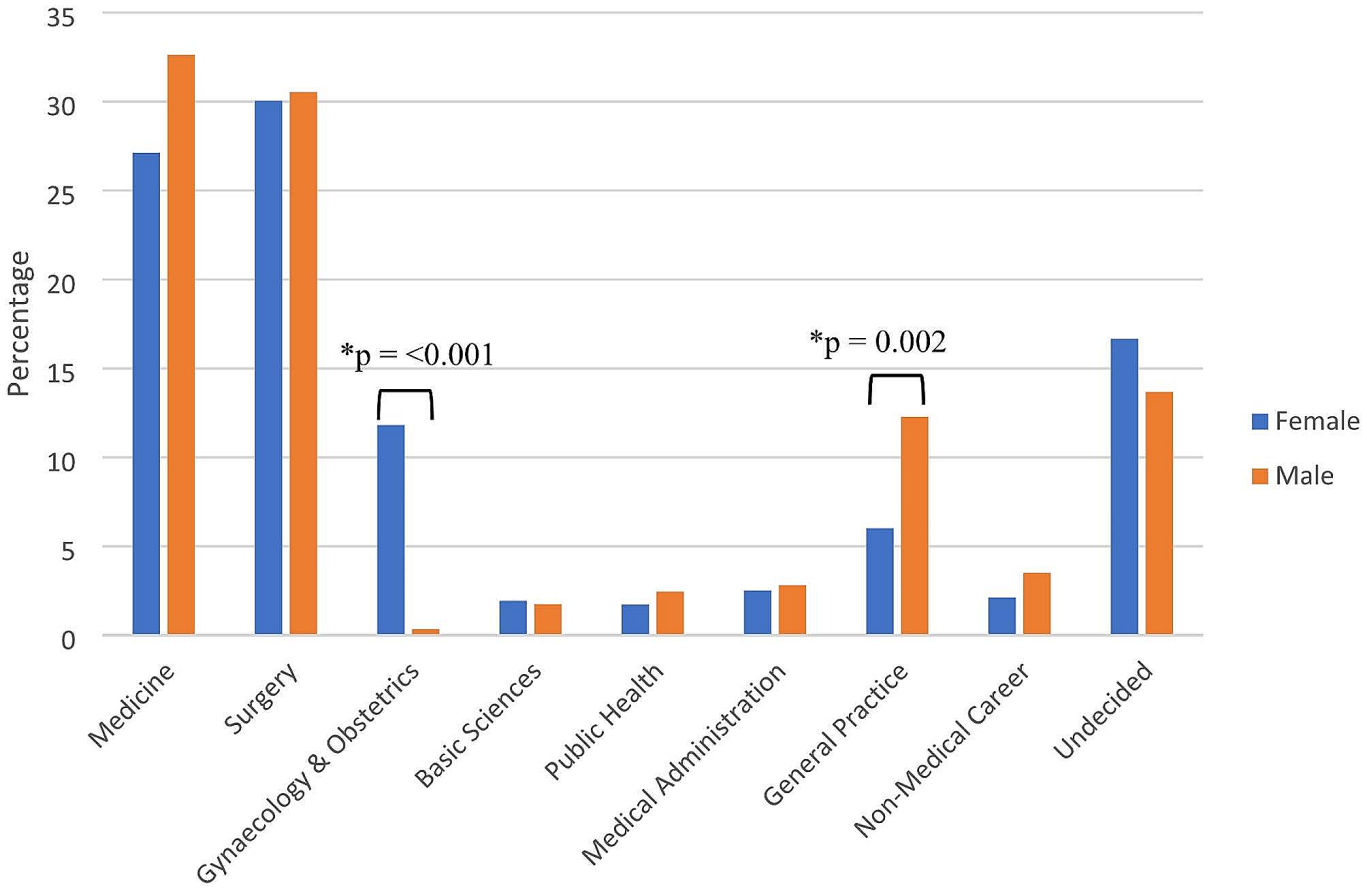
The comparison of first-choice career preferences by different genders among the medical students of Bangladesh

Figure 2 shows the comparison of first-choice career preferences by gender among the medical students of Bangladesh during the 2022 academic year. In comparison to male medical students, a significantly higher proportion of female medical students (*p* < 0.001) choose gynaecology & obstetrics. On the other hand, male medical students had a significantly higher tendency to choose general practice as a career than female medical students (*p* = 0.002). Furthermore, male students are more interested in medicine than female students, whereas female students showed more undecided trends in terms of future career choices.

**Fig. 3:**
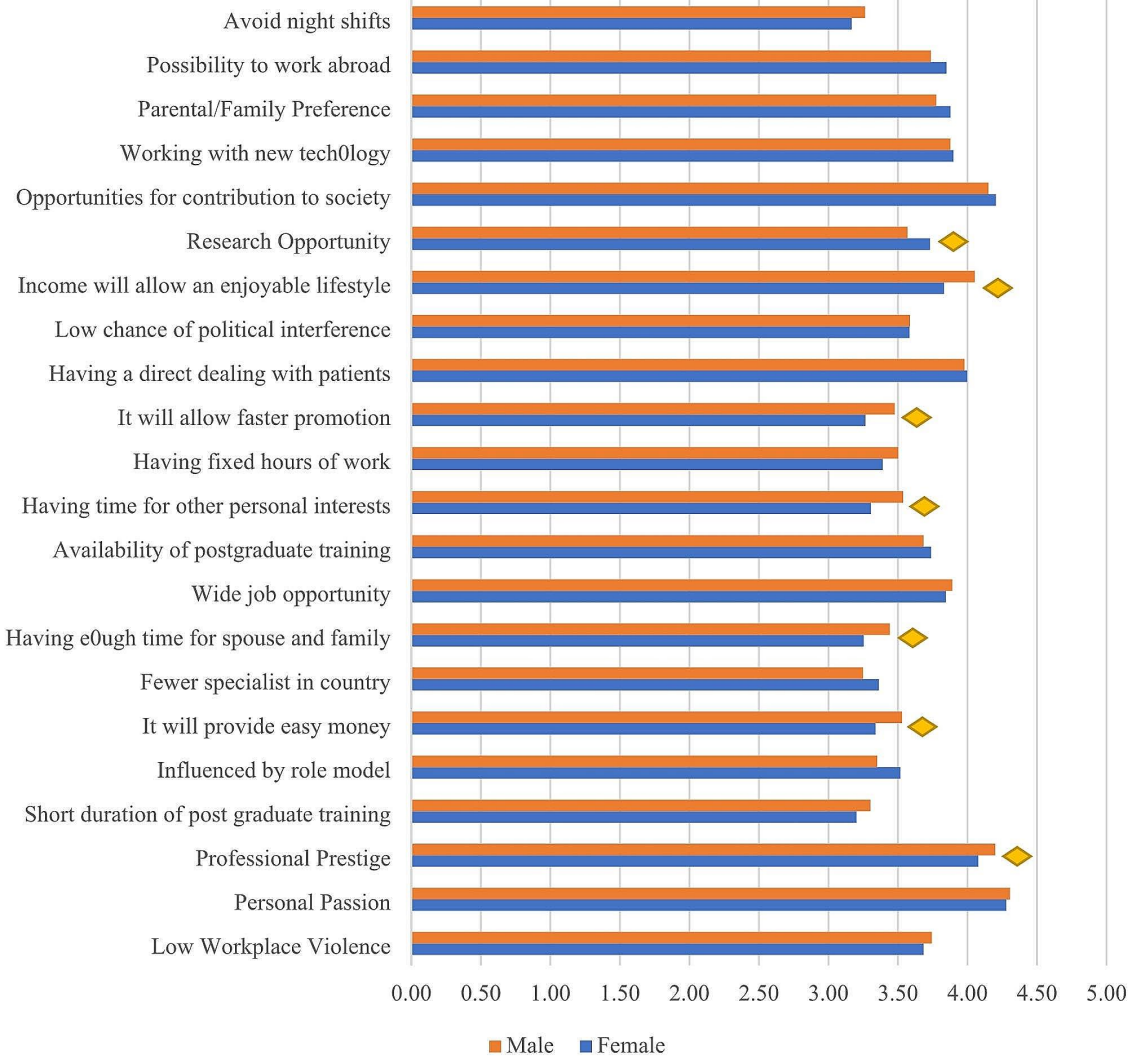
Comparison of factors affecting the career preference of study participants by gender (*n* = 801). Responses were based on the mean score from a 5-point Likert-type scale with strongly disagree = 1, disagree = 2, neutral = 3, agree = 4, and strongly agree = 5. Mann-Whitney U test was used to determine the differences between male and female students. A *p*-value of < 0.05 are presented as yellow diamonds

The comparison of factors affecting the career preference of study participants by gender are shown in Fig. 3. There is a significant difference between male and female students in their responses to factors such as professional prestige, being influenced by a role model, which will provide easy money, having enough time for spouse and family, having time for other personal interests, which will allow faster promotion, income will allow an enjoyable lifestyle, and research opportunity (*p* < 0.05).

### Academic year differences in career choice

**Fig. 4:**
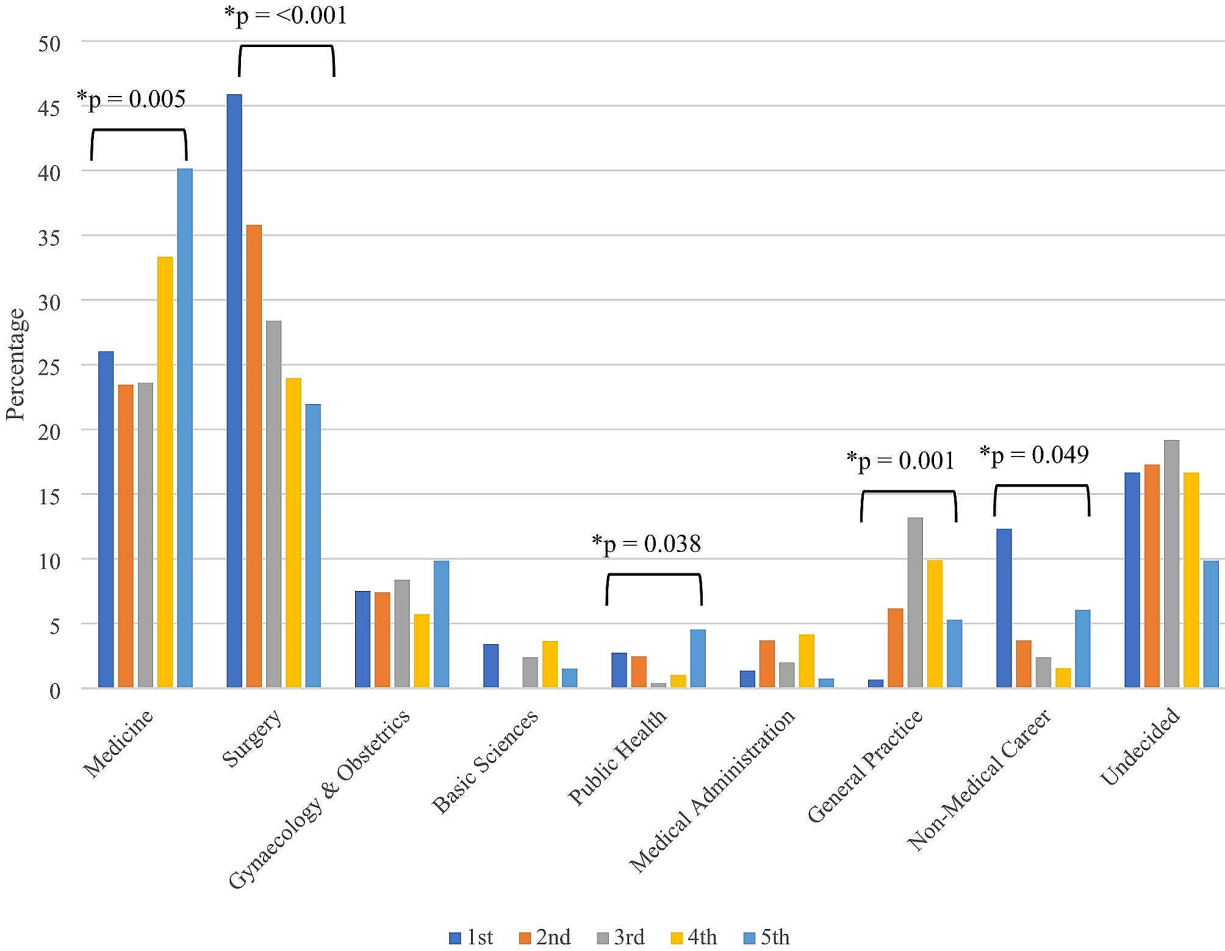
The comparison of first-choice career preferences by academic year among the medical students of Bangladesh during the 2022 academic year

In the field of medicine, the percentage of students intending to pursue a career in medicine and public health showed a significantly increasing trend over time (Fig. 4). Whereas, the percentage of students interested in surgery decreased significantly between 1st year to final year students (*p* < 0.001). Similarly, in general practice, the percentage of students interested in pursuing a career in this field increased significantly till the 3rd academic year and again decreased in the 5th academic year (*p* = 0.001). Furthermore, preference for non-medical careers decreased significantly in students of 5th academic year compared to the 1st year students (*p* < 0.05).

Table 3 presents the comparison of factors affecting the career preference of study participants by academic year. Overall, personal passion, opportunities for contribution to society, professional prestige, having a direct dealing with patients, and income will allow an enjoyable lifestyle were the most important factors in the choice of their career. Significant differences were found in the selection of future career options between the students of different academic years regarding low workplace violence, professional prestige, influenced by role models, fewer specialists in the country, fixed working hours, research opportunities, opportunities for contribution to society, working with new technology enjoyable lifestyle, and having enough time for spouse and family (*p* < 0.05).

**Table 3:** Comparison of factors affecting the career preference of study participants by academic year (*n* = 801)

Factors	Overall	Academic Year					*p*-value
		1st	2nd	3rd	4th	5th	
Low Workplace Violence	3.71	3.53	3.70	3.80	3.82	3.56	**0.041**
Personal Passion	4.29	4.21	4.20	4.33	4.28	4.36	0.635
Professional Prestige	4.12	4.19	4.31	4.11	3.98	4.14	**0.026**
Short duration of post graduate training	3.24	3.18	3.30	3.32	3.26	3.08	0.220
Influenced by role model	3.46	3.29	3.57	3.65	3.34	3.39	**0.011**
It will provide easy money	3.41	3.22	3.54	3.43	3.51	3.35	0.084
Fewer specialist in country	3.32	3.62	3.37	3.42	3.10	3.11	**0.000**
Having enough time for spouse and family	3.32	3.11	3.54	3.28	3.52	3.20	**0.008**
Wide job opportunity	3.86	3.89	4.04	3.90	3.73	3.83	0.243
Availability of postgraduate training	3.72	3.64	3.88	3.72	3.70	3.74	0.544
Having time for other personal interests	3.39	3.18	3.52	3.44	3.43	3.37	0.098
Having fixed hours of work	3.43	3.16	3.43	3.55	3.51	3.38	**0.016**
It will allow faster promotion	3.34	3.27	3.42	3.46	3.27	3.26	0.322
Having a direct dealing with patients	3.99	4.01	4.14	3.98	3.85	4.10	0.111
Low chance of political interference	3.58	3.48	3.69	3.65	3.55	3.56	0.573
Income will allow an enjoyable lifestyle	3.91	3.72	4.12	3.93	3.97	3.86	**0.023**
Research Opportunity	3.67	3.77	3.83	3.78	3.51	3.51	**0.005**
Opportunities for contribution to society	4.18	4.35	4.32	4.19	3.96	4.23	**0.001**
Working with new technology	3.89	3.95	3.99	4.02	3.69	3.81	**0.006**
Parental/Family Preference	3.84	4.05	3.89	3.87	3.68	3.77	0.068
Possibility to work abroad	3.81	3.67	3.98	3.88	3.69	3.90	0.140
Avoid night shifts	3.20	3.14	3.06	3.28	3.23	3.16	0.494

*Kruskal-Wallis H test was used to determine the differences between students of different academic years

^†^Significant *p*-values are in bold

Responses were based on the mean score a 5-point Likert-type scale with strongly disagree = 1, disagree = 2, neutral = 3, agree = 4, and strongly agree = 5

## Discussion

This study investigated the career preferences of 801 medical students in Bangladesh.

Our results show, majority of the students’ (65%) overall preference was medicine, while surgery was the most popular ‘first’ choice (30.21%) among the participants. The least preferred career choices were non-clinical such as basic sciences, public health, medical administration, etc. These findings are consistent with two studies conducted in India and Pakistan [17, 18] which showed inclination of undergrad medical students towards medicine and surgery.

Our findings indicate that female medical students exhibit a greater predisposition to gynecology & obstetrics as compared to their male counterparts. This aligns with prior research studies conducted in India and Saudi Arabia revealing female medical students’ greater preference for gynecology and obstetrics than their male counterparts [19, 20]. while male students preferred general surgery and internal medicine [20]. The higher tendency of female medical students to choose gynecology and obstetrics as their future specialty can be attributed to various factors, including social norms, cultural beliefs and personal interests. For example, women are often socialized to be more nurturing and caring, advocating women’s health and comfortable and safe dealing with same gender populations may thus be drawn to specialties that involve caring for women’s health and reproductive needs.

Conversely, male students display a significantly higher proclivity towards general practice when compared to female medical students. A Scottish study reported that male medical students were more motivated to pursue surgery than general practice, whereas female students were equally or more likely to choose general practice [21]. However, evidence from past studies stated female students are more likely to choose general practice as their first choice than male students [22]. Our study revealed male students are more attracted to medicine than female students, while female students showed more undecided trends. This finding is similar as reported by several studies [18, 23, 24] attributing cultural norms constraining girls’ opportunities to pursue higher education. These findings highlight the need for medical education programs to provide career guidance and support to students, particularly to female students who may face more challenges in choosing and practising their preferred specialities after the graduation.

However, according to two studies conducted in Jordan and Kenya, male students are more inclined to choose surgery and internal medicine, and orthopaedics, whereas female students preferred obstetrics and gynaecology the most [25, 26]. Another study from China stated similar insights that surgery and orthopaedics were male-dominated specialities, whereas the choices of female students are considerably fluctuating such as internal medicine, ophthalmology, neurology, dermatology, radiology, anaesthesia, pathology, gynaecology and obstetrics, and paediatrics [27]. On the other hand, few studies conducted in England, Netherlands, and the USA reported both male and female students were equally interested in internal medicine [28–31, 23, 24].

Our research reveals some interesting trends in the career preferences over time among medical students in Bangladesh. One trend that stands out is the increasing interest in pursuing a career in medicine over time. This trend has also been observed in other studies, which suggests that medical students are becoming more attracted to the field of medicine [32–34]. This could be due to various factors, such as the growing demand for medicine due to more opportunities for private practice or more job opportunities, perceived income potential, the scope of attending a wide range of patients, lifestyle, prestige etc. Another trend observed in this study is the decreasing interest in pursuing a career in surgery over time. This observation is supported by another study conducted among medical students in Greece [35]. It may be due to the challenging and demanding nature of surgical careers, which may not be appealing to all medical students [35, 36]. On the contrary, some studies reported increased interest in general surgery over time [32–34].

The results of this study indicate that the interest of medical students in public health as a career option increases significantly in the 5th year [26]. A similar trend was also found in a previous study ( [26, 37]). The reason behind this could be high salaries and facilities in NGOs and INGOs [38], competitive post-graduation training position etc. [37]. Additionally, this study also highlights a decreasing interest in non-medical careers among medical students from 1st year to the final year, which is in line with the results of other studies [33, 34]. It may be due to increased interest in clinical practice due to more clinical exposure over the course of their study However, an opposite trend was observed in a South Korean study reporting a significant proportion of medical students considering non-medical careers, such as business administration, law, and finance, indicating a potential shift in career aspirations over time [39].

One important finding of this study is the medical student’s preference of non-health cadres in Bangladesh Civil Service (i.e., any cadre, administration cadre, foreign cadre, police cadre). The main reason behind this phenomenon is inter-cadre disparity [40]. The reason for this trend could be attributed to mandatory posting in rural areas, lack of resources, safety etc. [37]. In contrast, administrative jobs come with a personal assistant, a separate office, access to a car, and prospects for ongoing promotion [41, 42].

We observed that professional prestige, the influence of role models, the potential for financial gain, the ability to maintain a work-life balance, time for personal interests, opportunities for fast promotion, the income level for a desirable lifestyle, and the chance for research are some important considerations for medical students when choosing their future careers. In a study conducted in Iran, professional prestige was identified as one of the most important factors influencing career choice among medical students [43]. Similarly, the current study found that professional prestige was significantly different between academic years, with first-year students having the highest mean score and fourth-year students having the lowest mean score. Role models (i.e., favourite faculties, senior/ renowned doctors with national/ international recognitions) have also been identified as a significant factor in career choice for medical students in this present study. In a study conducted in Pakistan, medical students reported that observing and interacting with a role model had a significant influence on their career choice [44].

Having fixed hours of work, and having enough time for spouses and families were significant factors in the selection of future career options across gender and academic years. These factors are important in work-life balance [45] and a study found that work-life balance was one of the most important factors influencing career choice among medical students [46]. However, the current study’s findings also highlight some factors that did not show significant differences between academic years. For example, personal passion, availability of postgraduate training, short duration of a post-graduation course, and the possibility of working abroad did not show any significant differences between academic years. This finding is consistent with past research studies that have identified these factors as important considerations for medical students regardless of their academic year [25, 47].

The present study indicates a notable difference between male and female medical students in their responses to certain factors that influence their career preferences. Those factors are such as professional prestige, being influenced by a role model, which will provide easy money, having enough time for spouse and family, having time for other personal interests, which will allow faster promotion, income will allow an enjoyable lifestyle, and research opportunity, which is consistent with previous research studies. For instance, a study conducted by Querido et al. found that male and female medical students have differing perceptions and preferences on the importance of work-life balance and family responsibilities [48]. Additionally, Lambert et al. reported that female medical students were more likely than male students to take lifestyle factors into account when choosing a specialty [36]. However, a study found that male and female medical students did not differ in their preferences for certain specialties based on the factors like job security, intellectual challenge, and opportunities for research [35].

This study was a cross-sectional study, thus, causal inference between the outcome and independent variables cannot be demonstrated. Moreover, career preferences can be changed over time. Due to the small sample size, the findings may not be representative of all medical students throughout the country. Further studies including all medical colleges of Bangladesh are recommended to draw conclusions. Another limitation of this study is that we did not follow up with students, so, the reasons for changes in career preferences throughout the course cannot be established. Despite some limitations, this study highlighted some important insights into the medical student’s future career choice pattern. The inclusion of medical students from all five academic years is also another strength of this study. The findings from this study will guide further studies involving all medical colleges of Bangladesh to understand the reasons for evolving medical students’ career choices.

## Conclusion

The findings of this study provide valuable insights into the career preferences and factors influencing the choices among medical students in Bangladesh. Medicine specialty was overall the most popular career choice, and surgical specialty being the most popular first choice. General practice, and gynaecology and obstetrics was the significantly popular choice among male and female students, respectively. Personal passion, opportunities for contribution to society, professional prestige, having a direct dealing with patients, and income will allow an enjoyable lifestyle were the most important factors in the choice of their career. Professional prestige, research opportunity, providing easy money, having enough time for spouse and family, and having sufficient income enjoyable lifestyle were the factors that showed significant gender difference in career choice for medical students. Understanding these factors can help medical educators and policymakers to design appropriate strategies that meet the needs and aspirations of medical students and support their future career development.

### Electronic supplementary material

Below is the link to the electronic supplementary material.

Supplementary Material 1

## Data Availability

The dataset used and/or analysed during the current study are available from the corresponding author Dr. Mohammad Azmain Iktidar on reasonable request, at sazmain@gmail.com.
