# Correction: Medical students’ career preferences in Bangladesh

**DOI:** 10.1186/s12909-024-05189-5

**Published:** 2024-02-28

**Authors:** Mohammad Azmain Iktidar, Md Muid Sakib, Ummi Rukaiya Munni, Fahmida Hoque Rimti, Renessa Yousuf, Koushik Majumder, Tirtha Saha, Farhat Lamisa Golpo, Md Samee U Sayed, Sabrina Monsur, Asadul Al Galib, Md Kamran Hossain, Sigma Alam Shupti, Noshin Nawar, Sudeshna Mazumder, M. Tasdik Hasan

**Affiliations:** 1 Directorate General of Health Services, Dhaka, Bangladesh; 2 Department of Public Health, North South University, Plot # 15, Block # B, Bashundhara R/A, 1229 Dhaka, Bangladesh; 3 School of Research, Chattogram, Bangladesh; 4 Chattogram Medical College, 57 K.B. Fazlul Kader Road, 4203 Chattogram, Bangladesh; 5 International Centre for Diarrhoeal Disease Research, Dhaka, Bangladesh; 6 Rajshahi Medical College and Hospital, 6000 Rajshahi, Bangladesh; 7 Dhaka Medical College Hospital, 1000 Ramna, Dhaka, Bangladesh; 8 Sir Salimullah Medical College and Mitford Hospital, 1100 Mitford, Dhaka, Bangladesh; 9 IBN Sina Medical College and Hospital, 1216 Kallyanpur, Dhaka, Bangladesh; 10 Mymensingh Medical College and Hospital, Chorpara, Mymensingh Bangladesh; 11 University of Chittagong, Chattogram, Bangladesh; 12 Action Lab, Department of Human Centred Computing, Faculty of Information Technology, Monash University, Melbourne, Australia; 13 Department of Public Health, State University of Bangladesh (SUB), Dhaka, Bangladesh; 14 Public Health Foundation, Bangladesh (PHF, BD), Dhaka, Bangladesh

**Correction: BMC Medical Education (2024) 24:81**

10.1186/s12909-024-05050-9

Following publication of the original article [1], that the authors identified an error in the author name of Sudeshna Mazumder.

The incorrect author name is: Sudeshna Majumder

The correct author name is: Sudeshna Mazumder

The original article has been corrected.
