# Supplementary material for: Medical students’ career preferences in Bangladesh
Source: BMC Med Educ. 2024 Jan 23;24:81. doi: 10.1186/s12909-024-05050-9 (PMC10804597; doi:10.1186/s12909-024-05050-9)
Supplement: Supplementary file 1 — Supplementary Material 1 [file 12909_2024_5050_MOESM1_ESM.docx]

**Questionnaire**
**Medical students’ career preferences in Bangladesh**

Respondent. ID: Date of Interview: DD/MM/ YYYY

Name of Respondent:

Name of Medical College:

Mobile Number/E-mail of Respondent:

| **Section 1: Background Characteristics** | |
| --- | --- |
| 1.1 Medical College: ☐ Government ☐ Private | |
| 1.2 Medical College Location: ☐ Dhaka ☐ Chattogram ☐ Rajshahi ☐ Khulna ☐ Barisal ☐ Sylhet ☐ Mymensingh ☐ Rangpur | |
| 1.3 Academic Year: ☐ 1st ☐ 2nd ☐ 3rd ☐ 4th ☐ 5th | |
| 1.4 Age (in full years): XX years | 1.5 Gender: ☐ Male ☐ Female ☐ Other |
| 1.6 Religion: ☐ Islam ☐ Sanatan ☐ Buddhism ☐ Chiristianity ☐ Other | |
| 1.7 Marital Status: ☐ Ever Married ☐ Unmarried | |
| 1.8 Parent’s Highest Education: ☐ Illiterate ☐ Primary ☐ SSC ☐ HSC ☐ Bachelor ☐ Masters ☐ Doctoral | |
| 1.9 Any doctor first degree relative: ☐ Yes ☐ No | |
| 1.10 Monthly Income (BDT): ☐ <14,999 ☐ 15,000-29,999 ☐ 30,000-44,999 ☐ 45,000-59,999 ☐ >60,000 | |
| 1.11 Residence: ☐ Dhaka ☐ Chattogram ☐ Rajshahi ☐ Khulna ☐ Barisal ☐ Sylhet ☐ Mymensingh ☐ Rangpur | |
| 1.12 Number of Supplementary Examination: ……. | |

| **Section 2: Career Preference** | | | |
| --- | --- | --- | --- |
| 2.1 | First Choice | ☐ General Physician ☐ Medical Administration ☐ Medicine Specialty ☐ Surgical Specialty ☐ Gynecology & Obstetrics Specialty ☐ Basic Medical Subject ☐ Preventive & Social Medicine/Public Health ☐ Research ☐ Not Decided ☐ Non-medical career ☐ Other …………… | |
| 2.2 | Second Choice | ☐ General Physician ☐ Medical Administration ☐ Medicine Specialty ☐ Surgical Specialty ☐ Gynecology & Obstetrics Specialty ☐ Basic Medical Subject ☐ Preventive & Social Medicine/Public Health ☐ Research ☐ Not Decided ☐ Non-medical career ☐ Other …………… | |
| 2.3 | Third Choice | ☐ General Physician ☐ Medical Administration ☐ Medicine Specialty ☐ Surgical Specialty ☐ Gynecology & Obstetrics Specialty ☐ Basic Medical Subject ☐ Preventive & Social Medicine/Public Health ☐ Research ☐ Not Decided ☐ Non-medical career ☐ Other …………… | |
| 2.4 | If you chose to leave medical career, which non-medical career would you choose? | |  |
| ***General practice*** *(provide comprehensive primary healthcare to individuals and families)****,*** ***medical administration*** *(management and coordination of healthcare facilities and services)****,*** ***medicine specialty*** *(focus on the diagnosis and non-surgical treatment of various medical conditions)****,*** ***surgical specialty*** *(specialize in performing surgical procedures to treat a wide range of medical conditions)****,*** ***gynaecology & obstetrics specialty*** *(focus on women's reproductive health, addressing issues such as reproductive system disorders and pregnancy-related concerns, childbirth, and postpartum care)****,*** ***basic medical science*** *(encompasses areas like anatomy, physiology, biochemistry, pathology, microbiology and pharmacology)****,*** ***preventive & social medicine/public*** ***health*** *(concentrate on preventing diseases and promoting health at the community and population levels)****,*** ***research*** *(involve in the systematic investigation of medical phenomena to advance scientific knowledge)* | | | |

| **Section 3: Please tell us if following are the factors that influence your career preference:** | | |
| --- | --- | --- |
| 3.1 | Low Workplace Violence | ☐ Strongly Disagree ☐ Disagree ☐ Neutral ☐ Strongly Agree ☐ Agree |
| 3.2 | Personal Passion | ☐ Strongly Disagree ☐ Disagree ☐ Neutral ☐ Strongly Agree ☐ Agree |
| 3.3 | Professional Prestige | ☐ Strongly Disagree ☐ Disagree ☐ Neutral ☐ Strongly Agree ☐ Agree |
| 3.4 | Short duration of post graduate training | ☐ Strongly Disagree ☐ Disagree ☐ Neutral ☐ Strongly Agree ☐ Agree |
| 3.5 | Influenced by role model | ☐ Strongly Disagree ☐ Disagree ☐ Neutral ☐ Strongly Agree ☐ Agree |
| 3.6 | It will provide easy money | ☐ Strongly Disagree ☐ Disagree ☐ Neutral ☐ Strongly Agree ☐ Agree |
| 3.7 | Fewer specialist in country | ☐ Strongly Disagree ☐ Disagree ☐ Neutral ☐ Strongly Agree ☐ Agree |
| 3.8 | Having enough time for spouse and family | ☐ Strongly Disagree ☐ Disagree ☐ Neutral ☐ Strongly Agree ☐ Agree |
| 3.9 | Wide job opportunity | ☐ Strongly Disagree ☐ Disagree ☐ Neutral ☐ Strongly Agree ☐ Agree |
| 3.10 | Availability of postgraduate training | ☐ Strongly Disagree ☐ Disagree ☐ Neutral ☐ Strongly Agree ☐ Agree |
| 3.11 | Having time for other personal interests | ☐ Strongly Disagree ☐ Disagree ☐ Neutral ☐ Strongly Agree ☐ Agree |
| 3.12 | Having fixed hours of work | ☐ Strongly Disagree ☐ Disagree ☐ Neutral ☐ Strongly Agree ☐ Agree |
| 3.13 | It will allow faster promotion | ☐ Strongly Disagree ☐ Disagree ☐ Neutral ☐ Strongly Agree ☐ Agree |
| 3.14 | Having a direct dealing with patients | ☐ Strongly Disagree ☐ Disagree ☐ Neutral ☐ Strongly Agree ☐ Agree |
| 3.15 | Low chance of political interference | ☐ Strongly Disagree ☐ Disagree ☐ Neutral ☐ Strongly Agree ☐ Agree |
| 3.16 | Income will allow an enjoyable lifestyle | ☐ Strongly Disagree ☐ Disagree ☐ Neutral ☐ Strongly Agree ☐ Agree |
| 3.17 | Research Opportunity | ☐ Strongly Disagree ☐ Disagree ☐ Neutral ☐ Strongly Agree ☐ Agree |
| 3.18 | Opportunities for contribution to society | ☐ Strongly Disagree ☐ Disagree ☐ Neutral ☐ Strongly Agree ☐ Agree |
| 3.19 | Working with new technology | ☐ Strongly Disagree ☐ Disagree ☐ Neutral ☐ Strongly Agree ☐ Agree |
| 3.20 | Parental/Family Preference | ☐ Strongly Disagree ☐ Disagree ☐ Neutral ☐ Strongly Agree ☐ Agree |
| 3.21 | Possibility to work abroad | ☐ Strongly Disagree ☐ Disagree ☐ Neutral ☐ Strongly Agree ☐ Agree |
| 3.22 | Avoid night shifts | ☐ Strongly Disagree ☐ Disagree ☐ Neutral ☐ Strongly Agree ☐ Agree |
| *The responses will be scored on a 5-point Likert-type scale with* *Strongly Disagree = 1, Disagree =2, Neutral = 3, Agree = 4, and Strongly Agree = 5* | | |
